# Mr. Crowfoot's Case of Burn

**Published:** 1805-01-01

**Authors:** W. Crowfoot

**Affiliations:** Beccles


### *To the Editors of the Medical and Physical Journal.*

Gentlemen,

Be pleased to give the following a place in your Journal if you think it of sufficient interest to deserve publication. The treatment, you will observe, was according to the improved practice of Dr. Kentish.

A young gentleman had incautiously left a pound of gunpowder in an open paper, and, forgetful of the circumstance, was examining the lock of his pistol; a spark from which, upon pulling down the flint, set the whole on fire, and enveloped him instantly in the flame and smoke, so that he was utterly at a loss to find his way out of the room; very providentially, however, the accident was immediately mediately discovered, and some of the family speedily extinguished the fire upon his cloaths; an *under flannel dress* had preserved the trunk from injury, though the linen waistcoat and shirt were burnt through in several places. The head and face had suffered severely, and I found him in pain to a degree of agony, complaining of the burning sensation in his face, and begging for some *cold application* to relieve him.

After covering the injured part with spirit of turpentine, I gave him thirty drops of laudanum in a glass of wine, and in ten minutes (as he continued in extreme pain) twenty drops more, and recommended he should be put into a warm bed, for he complained of general coldness, and was in the most compleat alarm as to the consequence. His attendant was directed to give twenty drops of the laudanum, in addition to what he had taken, if the pain did not abate in half an hour; but there was no occasion for the repetition of the anodyne, he soon became perfectly easy, and passed an exceeding good night. Several vesications appeared on the neck and throat the next morning, but these healed readily; and after a few days confinement to his room, and a redness of the whole face, which continued for several weeks, he got well without any mark or blemish whatever from the burn.

I have not thought it necessary to be very minute in detailing this case, as the object intended is merely to appreciate the sound and philosophical principles upon which the practice is founded. I have not seen any paper upon this interesting and important subject in your Journal; and if it directs the attention of medical men to the valuable pamphlet of Dr. K. I shall have the satisfaction of paying a just compliment to the merit of the author, and serving at the same time, as he proposed, the cause of humanity.

The Editors would perform a very acceptable service to the community if they would condescend to add their strictures upon the present fashionable dress of light and inflammable materials; pointing out the injurious effects upon the health in a general view, and the almost certain destruction of the individual in the event of any accident by fire.

I am, &c.

W. CROWFOOT.

